# Mitigating Latent Threats Identified through an Embedded *In Situ* Simulation Program and Their Comparison to Patient Safety Incidents: A Retrospective Review

**DOI:** 10.3389/fped.2017.00281

**Published:** 2018-02-01

**Authors:** Philip Knight, Helen MacGloin, Mary Lane, Lydia Lofton, Ajay Desai, Elizabeth Haxby, Duncan Macrae, Cecilia Korb, Penny Mortimer, Margarita Burmester

**Affiliations:** ^1^Paediatric Intensive Care Unit (PICU), Royal Brompton and Harefield NHS Trust, London, United Kingdom; ^2^Paediatric Anaesthesia, Royal Brompton and Harefield NHS Trust, London, United Kingdom; ^3^Risk Management, Royal Brompton and Harefield NHS Trust, London, United Kingdom

**Keywords:** patient safety, incident reporting and analysis, quality improvement, education, simulation, *in situ* characterization

## Abstract

**Objective:**

To assess the impact of service improvements implemented because of latent threats (LTs) detected during *in situ* simulation.

**Design:**

Retrospective review from April 2008 to April 2015.

**Setting:**

Paediatric Intensive Care Unit in a specialist tertiary hospital.

**Intervention:**

Service improvements from LTs detection during *in situ* simulation. Action plans from patient safety incidents (PSIs).

**Main outcome measures:**

The quantity, category, and subsequent service improvements for LTs. The quantity, category, and subsequent action plans for PSIs. Similarities between PSIs and LTs before and after service improvements.

**Results:**

201 Simulated inter-professional team training courses with 1,144 inter-professional participants. 44 LTs were identified (1 LT per 4.6 courses). Incident severity varied: 18 (41%) with the potential to cause harm, 20 (46%) that would have caused minimal harm, and 6 (13%) that would have caused significant temporary harm. Category analysis revealed the majority of LTs were resources (36%) and education and training (27%). The remainder consisted of equipment (11%), organizational and strategic (7%), work and environment (7%), medication (7%), and systems and protocols (5%). 43 service improvements were developed: 24 (55%) resources/equipment; 9 (21%) educational; 6 (14%) organizational changes; 2 (5%) staff communications; and 2 (5%) guidelines. Four (9%) service improvements were adopted trust wide. 32 (73%) LTs did not recur after service improvements. 24 (1%) of 1,946 PSIs were similar to LTs: 7 resource incidents, 7 catastrophic blood loss, 4 hyperkalaemia arrests, 3 emergency buzzer failures, and 3 difficulties contacting staff. 34 LTs (77%) were never recorded as PSIs.

**Conclusion:**

An *in situ* simulation program can identify important LTs which traditional reporting systems miss. Subsequent improvements in workplace systems and resources can improve efficiency and remove error traps.

## Introduction

Simulation is an important method for improving patient safety and quality of care (1) at individual, team, and system level (2). Team-based simulation can improve patient safety (3–8) by targeting team deficiencies, the greatest driver of lapses in patient safety (9). However, teamwork training alone will not overcome flaws in the fabric of the workplace. High reliability organizations (e.g., civil aviation) use *in situ* simulation to expose system vulnerability and hidden system flaws. These hidden threats are termed latent threats (LTs) (10). Proactive LT detection by *in situ* simulation is reported in emergency medicine, anesthesia, and pediatrics (11–13). *In situ* simulation is ideal for identifying LTs in the real work environment, using actual hospital systems, particularly during infrequent high stakes circumstances (14). Vulnerabilities in delivery settings deemed “safe” have been unveiled (11) and flaws in facility operational effectiveness detected (13, 15). *In situ* simulation offers a systematic and realistic picture of work because system flaws are contextualized in real time and place (3).

Patient safety incidents (PSIs) are unintended events with potential for patient harm, and all members of staff are encouraged to report these to Datix^®^. PSI reporting is a statutory function of National Health Service (NHS) Improvement to reduce patient risks through the National Patient Safety Alerting System (16). Critiques of PSI reporting highlight a misplaced scrutiny on counting reports, rather than organizational learning from events (17, 18) and the difficulties of feeding-back to frontline staff (19–21).

There is a paucity of research on the effect of LT detection on traditional safety models such as Datix^®^.

We hypothesized that LTs identified during *in situ* simulation courses could occur in real life; therefore, mitigating LTs through service improvements might improve patient safety. We aimed to measure:

1. The quantity, category, and subsequent service improvements of LTs.
2. The quantity, category, and subsequent action plans for PSIs (reported on Datix^®^) occurring during the same period and including 1 year before the program inception in 2008.
3. The similarities between PSI Datix^®^ and LTs before and after service improvements.

## Materials and Methods

The Simulated interPRofessional Team Training (SPRinT) program at Royal Brompton Hospital is an embedded *in situ* inter-professional simulation team training program that runs courses in all areas of pediatric care (22). SPRinT faculty are frontline multidisciplinary staff including nurses, nurse educators, anesthetic and intensive care consultants, and medical and surgical trainees. There is a minimum of one nurse and one doctor faculty member at each SPRinT course. Participants are all staff involved with pediatric care including all grades of medical, surgical, and anesthetic staff (consultant and junior doctors), nursing staff (charge nurse and band five to seven nurses), and allied health professionals (health-care assistants, family liaison officers, and physiotherapists). The validated *in situ* 2-h SPRinT session (23) includes crisis resource management training, a simulated scenario followed by 45 min video-assisted debriefing [utilizing advocacy–inquiry methodology (24)]. Scenarios are derived from real events including serious untoward incidents. Identification, investigation, and mitigation of LTs uncovered during courses are an integral part of SPRinT’s commitment to improving patient safety.

### LTs and Service Improvements

During the debriefing after a simulation scenario, all faculty and participants identify any LTs that have occurred and suggest and discuss proposals for service improvement. These events are recorded at the time by two faculty members on a specific LT *pro forma*. LT severity is graded according to national Datix^®^ coding system. Datix^®^ is the online reporting system used by NHS organizations to report PSIs. They color code incidents from potential to cause harm (green), to causing minimal harm (yellow), to causing significant temporary harm (amber), and to permanent harm or death (red). LTs were uploaded to a SPRinT risk database from 2008 until February 2012 after which a specific LT reporting section on Datix^®^ was developed, the reporting criteria remained the same.

Latent threats recorded from Paediatric Intensive Care Unit (PICU) SPRinT courses from April 2008 to April 15 were retrospectively reviewed. SPRinT clinical fellows PK and HM independently categorized LTs according to seven descriptive categories: education and training, resources, equipment, work and environment, medication, organization and strategic, and protocols and systems. All differences were resolved by agreement.

Service improvements for LTs discussed during the SPRinT debriefing session are implemented by the SPRinT and department lead. All service improvements were quantified and grouped independently (HM and PK) according to nine categories: no change; looking into; ongoing quality improvement project (QIP); staff communication; guidelines; education; equipment/resources; organizational change; and all events reviewed by root cause analysis, serious case review, or serious hazard of transfusion.

### PSIs and Action Plans

All PSIs on PICU reported to Datix^®^ from April 2007 to April 2015 were reviewed and independently categorized by Philip Knight and Helen MacGloin according to the same seven descriptive categories used for LTs. All differences were resolved by agreement.

Hospital risk management, quality, and safety departments organize regular review of Datix^®^ and subsequent action plans are made. These action plans are instituted by the lead for incident reporting for that department. Action plans for PSIs were quantified and grouped independently (Helen MacGloin and Philip Knight) according to the same nine categories used for service improvements.

### Statistical Comparison

The quantity, category, and grading of LTs and PSIs were compared to assess any impact of LT service improvements on PSIs over time. This review was registered as a quality improvement initiative exempt from ethics review.

## Results

### Category Analysis of PSIs and Their Action Plans

1,946 PSIs were identified with severity grading of 1,405 (72%) with the potential to cause harm (green), 480 (25%) causing minimal harm (yellow), 60 (3%) causing significant temporary harm (amber), and 1 (0.05%) causing permanent harm or death (red). 1,140 (59%) PSIs could not be categorized because the precipitating cause could not be accurately identified, e.g., unanticipated surgery, initiation of extracorporeal membrane oxygenation.

Of 806 (41%) PSIs that could be categorized, medication PSIs accounted for 512 (64%), most reported errors in drug recording or controlled drug spillages. Other medication PSIs included prescribing errors, delayed or incorrect administration, or issues with expired and out of stock medications. 133 (17%) resource PSIs included missing resuscitation ventilator equipment, or infusion sets. 103 (13%) equipment PSIs included equipment failure requiring servicing or replacement. 29 (4%) work and environment PSIs included issues with arrest buzzers, blocked sinks, and bed-spaces. 13 (2%) PSI systems and protocols reports included provision of specialist services out of hours. Nine (1%) education and training PSIs included lack of knowledge of guidelines. Organization and strategic PSIs accounted for seven (0.9%) including incidents due to emergency admission pathways.

984 (51%) of all PSIs had 1,008 action plans; 962 (49%) reported only the event (without action plan). 622 (62%) involved staff communication (including individual staff spoken to, reminders, emails, signs safety and medicine bulletins, and patient safety newsletters). 132 (13%) planned to look into the event further; 104 (10%) planned new resources or fixed equipment; 40 (4%) planned organizational changes; 32 (3%) planned educational workshops, induction, changes to training or focused teaching; 32 (3%) planned to address the event within an existing QIP 24 (2%) new/updated guidelines; 22 (2%) were root cause analyses, serious untoward incidents, and serious hazards of transfusion.

### Category Analysis of LTs and Service Improvements

**Figure 1:**
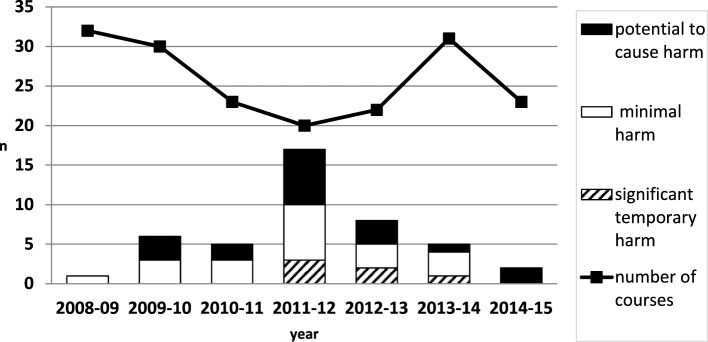
Latent threats over time.

201 SPRinT courses involved 1,144 inter-professional staff (488 doctors, 543 nurses, and 113 other allied professionals). 44 LTs were identified (1 LT per 4.6 courses) (Figure 1).

**Figure 2:**
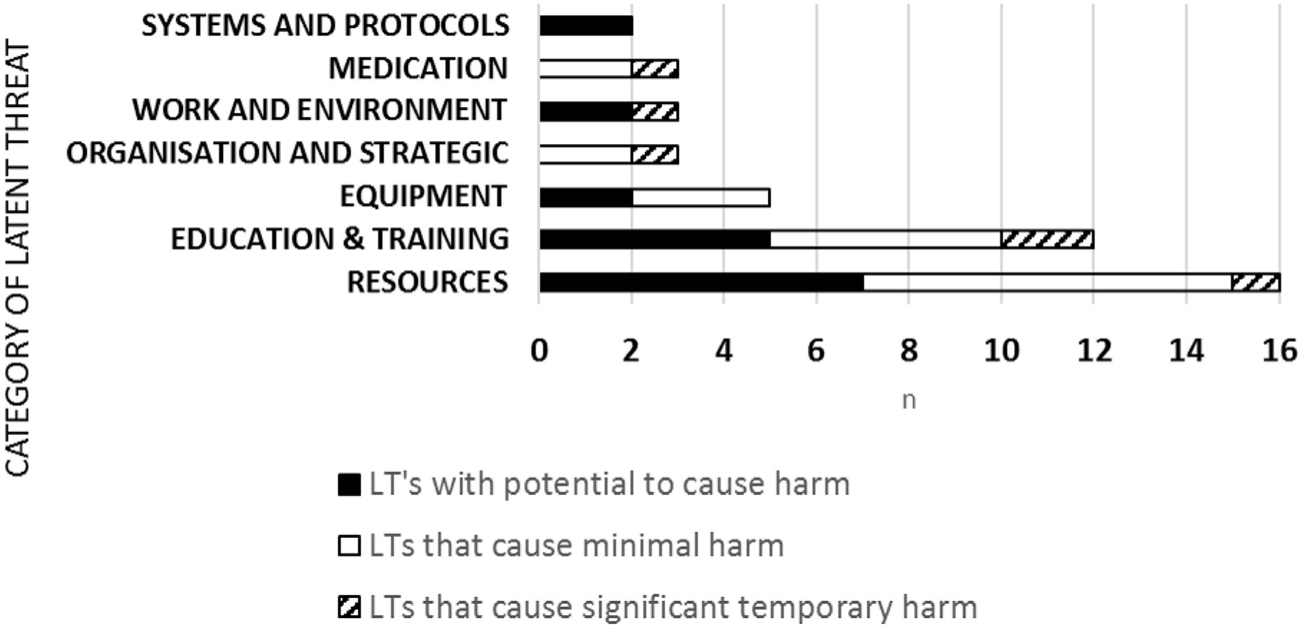
Latent threat category of risk.

44 LTs were uncovered and category analysis showed 16 (36%) related to resources, 12 (27%) education and training, 5 (11%) equipment, 3 (7%) organizational and strategic; 3 (7%) work and environment, 3 (7%) medication; 2 (5%) systems and protocols (Figure 2). LTs severity ranking of risk was 18 (40.9%) LTs with the potential to cause harm, 20 (45.5%) LTs that would cause minimal harm, 6 (13.6%) LTs that would cause significant temporary harm. LTs reached a peak in 2012 and then reduced over time year on year (Figure 1).

43 subsequent service improvements were developed (2 LTs addressed by one service improvement); 4 (9.3%) of which were implemented trust wide (Tables 1 and 2).

**Table 1:** LTs with the potential to cause harm.

LT with the potential to cause harm (*n* = 18)	LT category	System improvement	System improvement category
Emergency drug doses unknown without emergency drug chart	Resources	Patient-specific emergency drug chart printed before admission	Resources/equipment
No patient specific emergency drug chart available	Resources	Basic emergency drug doses chart added to arrest trolleys	Resources/equipment
Bleep numbers for on-call staff not known/easily accessible	Resources	Numbers for on-call teams displayed in all bays and PICU board	Resources/equipment
No lightweight in-line ETCO_2_ lines available—out of stock	Resources	Stock ordering change ensuring in-line CO_2_ lines available	Resources/equipment
Formula for sizing of ETT not known	Resources	ETT formula added to emergency drug chart for pre-calculation	Resources/equipment
Inability to identify pediatric from adult chest opening trolley	Equipment	Trolleys clearly labeled *(trust wide)*	Resources/equipment
ECG machine not available	Equipment	Bid for new ECG machine	Resources/equipment
ECG paper ran out during SVT scenario	Resources	Replenished and staff made aware of importance	Resources/equipment
Delay in finding magnets on arrest trolleys to reset ICD	Resources	Magnets added to arrest trolley contents	Resources/equipment
Staff unsure how to use magnets for resetting ICDs	Education and training	Workshops introduced	Education
New nursing staff did not know how to use emergency buzzers	Education and training	Wall buzzers labeled, nurse induction updated	Education
Radiographers not pediatric BLS trained	Education and training	BLS training for radiographers mandatory	Education
Nursing staff not EPLS/PILS trained	Education and training	Funding for additional places for EPLS and PILS training secured	Education
Nurse bleeped rather than dialing 2222 for crash call	Education and training	Email sent to all staff; 2222 instruction stickers on ward phones	Staff communication
Clock for timings during CPR difficult to see	Work and environment	Clocks moved to be more visible and provided in each side room	Resources/equipment
Buzzer did not sound when used in simulation	Work and environment	Buzzer system testing regularly	Organizational
CBL^a^ protocol difficult to follow	Systems and protocols	New CBL protocol and attached to arrest trolley (trust wide)	Guideline
Hyperkalemia guideline difficult to follow during emergency	Systems and protocols	Guideline adapted into easy to follow algorithm	Guideline

*LT, latent threat; PICU, Paediatric Intensive Care Unit; BLS, basic life support; EPLS, European Pediatric Life Support; ICDs, implantable cardioverter defibrillators; CPR, cardiopulmonary resuscitation; PSI, patient safety incident; CBL, catastrophic blood loss*.

*^a^LTs related to 2 CBL simulations annotated (*n* = 5)*.

*LTs never detected as PSI 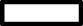
*.

*LTs reported as PSI 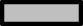
*.

**Table 2:** LTs that would cause minimal harm and significant temporary harm.

LT that would cause minimal harm (*n* = 20)	LT category	System improvement	System improvement category
Difficulty contacting PICU consultants *via* switchboard	Resources	Nurse in charge given PICU mobile with phone numbers	Resources/equipment
Intubation drugs in multiple areas—delaying intubation	Resources	Emergency intubation boxes introduced and stored in fridge	Resources/equipment
Intubation drugs in locked cupboard—delay in finding keys	Resources		
Doses for emergency intubation drugs not known	Resources	Dosage card developed for intubation box	Resources/equipment
Arrest algorithms not readily available during cardiac arrest	Resources	Arrest algorithms laminated and attached to arrest trolleys	Resources/equipment
Team unable to contact consultant cardiothoracic surgeon	Resources	Cardiothoracic phone numbers added to PICU phone	Resources/equipment
Lack of time awareness—delay in administering adenosine	Resources	Digital timers obtained for each arrest trolley	Resources/equipment
Naloxone dose and administration not known	Education and training	Naloxone added to patient-specific emergency drug chart	Resources/equipment
Staff^a^ unaware of CBL protocol	Education and training	CBL protocol workshops and emphasized at induction	Education
Staff unsure how to get additional help for deteriorating patient	Education and training	Email sent to all staff and reinforced at induction	Education
Staff reluctant to use pre-drawn up adrenaline	Education and training	Simulation nurse led educational drive on benefits/use	Education
Staff unfamiliar with item location in chest re-opening trolley	Education and training	Labels added to chest re-opening drawers	Resources/equipment
Wrong chest opening set on chest opening trolley	Equipment	Stocking of chest opening trolley reviewed	Organizational
Delay finding correct sized FM and T-piece post operatively	Equipment	All patients transferred with mask and T-piece from theater	Organizational
Suction unit for emergency chest re-opening trolley not working	Equipment	BME rectified defect with unit	Equipment
Adenosine not part of standard resuscitation drug tray	Medication	Adenosine added to resuscitation drug tray	Resources
Metaraminol unavailable during hypercyanotic scenario	Medication	Metaraminol added to resuscitation emergency drug tray	Resources
Difficulty^a^ contacting transfusion during a CBL scenario	Organizational	Emergency bleep for blood transfusion technician	Organizational
Cardiologist did not arrive with arrest team	Organizational	Pediatric cardiology registrar added to arrest team	Organizational
Over 3 min to find drug cupboard keys	Resources	Funding initiated for keyless drug cupboards	Resources/Equipment
**LT that would cause significant temporary harm (*n* = 6)**			
No insulin available for hyperkalemia leading to VF arrest	Medication	Ensured that insulin pharmacy requests in place	Resources/equipment
Echo unavailable when required urgently to confirm cardiac tamponade. Chest re-opened without echo confirmation due decompensating condition and subsequent cardiac arrest	Resources	Capital bid for new echo	Resources/equipment
Staff members did not know how to use defibrillator	Education and training	Defibrillator workshops introduced	Education
Emergency^a^ drug chart used to verify patient during CBL	Education and training	CBL protocol reinforced and increased emphasis at induction	Education
Staff^a^ unable to reach patient whilst phoning transfusion	Work and environment	Cordless phones obtained for emergency use	Resources/equipment
Wrong^a^ blood collected during CBL scenario	Organizational	Blood collection policy change—handed over person to person	Organizational

*LT, latent threat; PICU, Paediatric Intensive Care Unit; PSI, patient safety incident; CBL, catastrophic blood loss*.

*^a^LTs related to 2 CBL simulations annotated (*n* = 5)*.

*LTs never detected as PSI 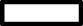
*.

*LTs reported as PSI 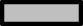
*.

There were three scenarios that were particularly high yield, uncovering multiple high-risk LTs. The first was a catastrophic blood loss (CBL) dual location SPRinT course in PICU and the Blood Transfusion Laboratory (BTL) involving three teams (anesthesia, PICU, and cardiothoracic surgery). Real-time simultaneous video-recording enabled a unique perspective on LTs at the PICU and the BTL interface. A 15-point PICU checklist was developed for this dual site simulation and its adherence was assessed (adherence 30%) and a 14-point checklist in BTL (adherence 100%). LT’s was uncovered including a delay contacting BTL technician, difficulty implementing emergency CBL management and collection of the wrong blood unit (Table 2). Following these events, emergency blood tracking requirements were changed so that issued blood units could be handed directly from BTL staff to PICU staff, rather than collection of blood units from the blood refrigerator.

Retesting of the CBL protocol during a second SPRinT course highlighted unfamiliarity with the new CBL protocol and failure to order all blood products (FPP, platelets, and cryoprecipitate). Subsequent service improvements included changes to mandatory staff training requirements, repeated simulation and refinement of CBL protocol with departmental education and subsequently implementation trust wide.

The second scenario involved an emergency resternotomy post cardiac surgery and uncovered five LTs that would have caused harm. They included the inability to perform a cardiac echocardiogram on a deteriorating simulated patient with cardiac tamponade, because the echo machine was in use elsewhere. Difficulty distinguishing adult and pediatric chest re-opening sets, difficulty in locating items on chest opening trolley, difficulty in contacting cardiothoracic surgeons, and delay in administering treatment due to time searching for drug cupboard keys (Table 2). Service improvements included a successful capital bid for an extra echo machine, purchase of mobile phone for nurse in charge and uploading consultants’ phone numbers, organizing automated drug cabinet and open chest trolley.

The third scenario was of hyperkalaemia progressing to ventricular fibrillation and cardiac arrest. LTs uncovered: lack of insulin (an essential part of emergency treatment), lack of awareness of the hyperkalaemia protocol, and lack of knowledge on correct use of the defibrillator. To mitigate the latter, *in situ* simulated electrophysiology workshops and defibrillation simulation scenarios reviewing external pacing and using internal defibrillation paddles were developed and 70 staff members participated (May 2011–February 2012).

### Impact of LT Service Improvements on Future PSIs

Latent threats matched 24 (1%) of 1,946 PSIs. The impact of service improvements for these LTs was investigated.

The LT of poor management of hyperkalaemia was preceded by four hyperkalaemia PSIs of different causes. After service improvement (Table 1), one PSI occurred which was a record of the hyperkalaemia event with appropriate treatment and no adverse event.

The emergency buzzer failure LT was preceded by three similar PSIs with planned follow-up by the operational manager. After service improvement of implementation of regular system testing (Table 1), there were no further PSIs.

The two LTs of difficulty calling essential staff to the simulated emergency were preceded by two similar PSIs. In 2009, simulation participants were unable to contact on-call PICU consultants *via* switchboard, following which a mobile phone programmed with all the PICU consultant contact details was obtained for the PICU charge nurse. However, difficulties contacting other essential staff resulted in the same LT recurring, therefore the mobile phone contact list was updated. No further LTs or PSIs from difficulties in calling essential staff recurred.

**Figure 3:**
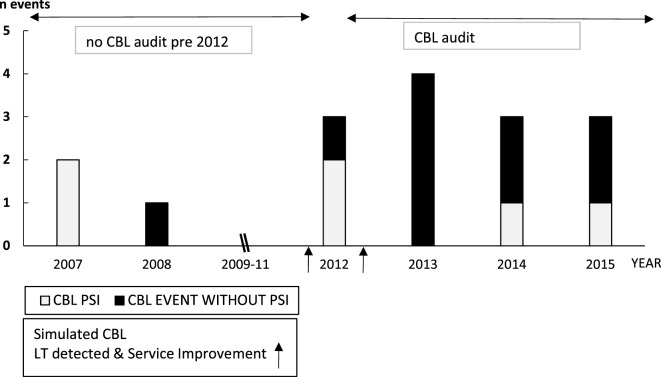
Paediatric Intensive Care Unit CBL Catastrophic Blood Loss Events (April 2007–2015) and impact of Simulation Led Service Improvements.

Three CBL PSIs preceded the first simulated CBL scenario (Figure 3) although before this, CBL was not audited; therefore, these data are not robust. One of these CBL PSIs highlighted poor team communication, recommending SPRinT simulations to improve team communication and emergency chest opening training, which was subsequently carried out.

Two CBL PSIs occurred between the first and second CBL simulations in 2012. The first CBL PSI reported incorrect patient identification resulting in transfusion with blood intended for another child. The second PSI reported incorrect activation of CBL protocol. LT system improvements were implemented subsequent to the second CBL simulation, and in 2013 four CBL events occurred without PSIs. CBL PSIs in 2014 and 2015 reported difficulties contacting the blood transfusion technician because a CBL arrest call had not been properly activated.

Resource LTs (ETCO_2_ lines and ECG paper) repeated as real events despite service improvements. Despite improved stock ordering after detecting lack of ETCO_2_ lines, this was followed by three similar PSIs. The LT from lack of ECG paper was preceded by one similar PSI and despite service improvement, recurred as two PSIs.

34 LTs (77%) were never recorded as PSIs therefore the impact of the relevant service improvement could not be assessed. This encompassed all systems and protocols LTs (2/2); all equipment LTs (5/5); 10/12 (83%) education LTs; 13/16 (81%) resource LTs; 2/3 (66.7%) work and environment LTs; 1/3 (33%) medication, and 1/3 (33%) organizational LTs.

## Discussion

*In situ* SPRinT courses identified LTs and implemented simple system adaptations rapidly with minimal cost or manpower. This echoes other *in situ* simulation studies reporting immediate risk mitigation and simple changes with few resources (12). *In situ* simulation coupled to system improvements is embraced by staff who witnessed the potential risk of LT to a real patient during simulation. Program engagement likely resulted from empowering staff to test hospital protocols, equipment, and environment for real system flaws. Such adaptive safety initiatives align with Berwick’s plea for frontline staff to be enabled to identify problems, test changes, and lead service improvements (25).

The majority of LTs were due to lack of resources and education and training deficits. Of the 12 LTs which recurred despite service improvements, half were due to lack of resources. Other LT studies similarly unveiled poorly understood policies, staff training deficiencies, and issues with critical materials and supplies (26, 27). In our review, seemingly improbable events during simulation (lack of insulin to treat hyperkalaemia and incorrect patient identification for blood transfusion) resurfaced in real life as PSIs. In addition, LTs uncovered significant training needs (e.g., defibrillation skills) unreported as PSI because education deficits are not well highlighted by incident reports.

Only 1% of PSIs were similar to LTs which might reflect the limitations of incident reporting and make PSIs poor measures of safety and quality improvement (28, 29). We also felt that because few of the LTs had corresponding PSIs this actually showed the unique value of simulation over patient safety reporting and are treating this as a positive finding. In addition, incident reporting and *in situ* simulation provide different vistas on safety. Other studies have highlighted the need for different approaches to build a comprehensive picture of safety because of the complementary yet different information provided by each approach (30).

Some repeat simulation scenarios enabled development and testing of protocols and a shared perspective ordinarily unavailable. For example, the dual location CBL simulations enabled a unique observation of simulated error at the interface of care between PICU and blood transfusion. This perspective resulted in an interdepartmental collaborative approach to patient safety, facilitated learning, and supported the rationale for system improvement. Although statistically impossible to prove CBL events are safer (due to rarity), CBL PSI grading lessened after system improvements. Likewise trust wide introduction of an intubation box reduced the time to intubate in all areas. Subsequently, excessive time to prepare for intubation has not recurred as an LT in simulated events. However, as never reported as a PSI, system improvement impact cannot be assessed, although it is obvious that reduction in time to intubate improves patient care in a high-risk environment.

Comparing medication LTs and PSIs is interesting. Although most PSIs reported medication errors, emergency drug dose calculations never featured as a PSI possibly because of one of the LT service improvements (emergency drug charts).

The traditional definition of safety (safety 1) is the absence of harm (31). However, safety is not just non-events but a proactive sensitivity to the possibility of failure (32). This deeper understanding of work-as-done enables anticipation of events and team and system flexibility and resilience (33). *In situ* simulation can encompass both safety perspectives. To the best of our knowledge, this is the first study to review LT detection over time, providing a rich reflection of work-as-done. Measuring LTs from simulation could be developed similarly by other trusts as a proxy safety metric, in conjunction with traditional retrospective PSI reporting, as complementary approaches to safety.

There were several limitations to this study. PSIs on Datix^®^ were reviewed to assess the impact of service improvements on safety. However, voluntary reporting of adverse incidents captures only a minority of incidents and is subject to significant bias (30). The possibility of bias and missing CBL events was recognized prompting review of the blood transfusion audit. PSI limitations could have been overcome by triangulating them with other records of patient safety events such as patient complaint records, risk management databases, and safety walk-rounds. Retrospective categorization was another source of potential bias. There is no standard nomenclature for error analysis on PICUs although frameworks for incident analysis could have been adapted (34). Other simulation studies have used failure modes effects analysis (FMEA) coupled to simulation training to prioritize predicted risk to enable solution development in stratified order (35). In contrast, we addressed LTs as detected, according to the perceived patient risk because some FMEA critiques highlight the lack of evidence base and the resource heavy methodology involved (36).

Iterative testing of system improvement efficacy was not performed which might have prevented later emergence of LTs as PSIs 2–3 years later. Follow-up interviews checking solution success might have enabled system improvement efficacy to be assessed (26). Another limitation was that the review was limited to PSIs and LTs from PICU, excluding LTs detected elsewhere in the directorate (e.g., PACU and the cardiac ward). Finally, participant psychological safety could have been threatened by reporting education and training LTs. Reassuringly, anonymous participant feedback never reported a negative impact on learning.

## Conclusion

To the best of our knowledge, this is the first study comparing LTs identified during *in situ* simulation to existing safety reporting systems and evaluate subsequent service improvements. The unique strengths of *in situ* simulation were highlighted. First, the identification of potential threats to patient safety particularly for training and knowledge gaps undetected elsewhere. Second, *in situ* simulation offered a real-time unbiased multi-professional approach to patient safety.

Modern health-care pressures require better models of safety (34) and perspectives reflecting health care’s socio-technical system complexity. This cannot be achieved by one safety model but the best way to measure their individual or combined impact is unclear.

More work is required to integrate the strengths of *in situ* simulation into traditional models of patient care and safety and robustly measure its efficacy. This would enable *in situ* simulation to be harnessed by existing health-care systems and accepted as a valuable safety improvement modality.

## Author Contributions

Participation in the conception and design of the work: MB, PK, and HM. Execution of the work: MB, LL, ML, CK, PK, AD, HM, EH, DM, and PM. Analysis of the data and contribution of methodological expertise MB, HM, and PK.

## Conflict of Interest Statement

The authors declare that the research was conducted in the absence of any commercial or financial relationships that could be construed as a potential conflict of interest. The reviewer EA and handling editor declared their shared affiliation.
